# Looking for social class in all the wrong places: differences in social support emerge reliably between—but not within—class contexts

**DOI:** 10.3389/fpsyg.2026.1688178

**Published:** 2026-07-01

**Authors:** Nicholas J. Fendinger, Siyi Lou, Eric D. Knowles

**Affiliations:** Department of Psychology, New York University, New York, NY, United States

**Keywords:** culture, culture cycle, latent profile analysis, LPA, social class

## Abstract

Despite theorizing that cultural phenomena reflect collective processes, psychologists often analyze such phenomena at the individual level. This approach can skew conclusions by obscuring processes that unfold between—rather than within—group contexts. To demonstrate the importance of studying cultural effects at the group level, we examine a key tenet of sociocultural models of social class: an inverse relationship between class and social support. Across two datasets (*N_total_* = 3,347), individual-level linear and nonlinear models revealed weak and inconsistent associations between class indicators and social support. However, when latent profile analysis (LPA) was used to model associations at the level of class *groups*, we reliably observed a working-class profile displaying higher levels of social support than a middle/upper-class profile. Corroborating the idea that poverty is associated with social isolation, we also identified an “underclass” profile combining extreme deprivation with low levels of social support. Our work underscores that class-cultural differences constitute group-level phenomena that may prove undetectable at the individual level.

## Introduction

Social class “is one of the most consequential social divides of our time” (Stephens et al., 2024, p. 2). As a material context, class influences individuals’ access to education (Grusky et al., 2019), healthcare (Chetty et al., 2016), housing (Massey, 2020), and jobs (Charles et al., 2019). As a cultural context that arises in response to material realities (Carey and Markus, 2017; Nisbett, 2003), social class shapes people’s psychologies in myriad ways—from cognitive styles (Grossmann and Varnum, 2011; Varnum et al., 2010), conceptions of agency (Stephens et al., 2011; Varnum et al., 2016), and interpersonal aptitudes (Dietze et al., 2024; Dietze and Knowles, 2016; Dietze and Knowles, 2021; Kraus and Keltner, 2009; Piff et al., 2012) to their educational motivations (Stephens et al., 2007, 2012), work preferences (Dittmann et al., 2020, 2024; Müller et al., 2023), and sociopolitical views (Kim et al., 2022; Schaffner et al., 2018).

“Social class” can be (and has been) defined in many ways. We take a sociocultural approach, according to which social classes locate individuals within a hierarchy of access to material, social, and symbolic resources required for thriving in a society (Grusky and Weeden, 2006; Stephens et al., 2024). Conceived in this way, social classes are multidimensional *contexts* (Bourdieu, 1986; Weber, 1920; Savage et al., 2013) that foster unique behavioral norms, values, lay theories, and self-construals—or *cultures* (Lamont, 2000; Lareau, 2003; Stephens et al., 2024). A class culture reflects mutually constitutive links between the resources available within a class context and the individual cognitions, preferences, and emotions evoked by those resource affordances (Carey and Markus, 2017; Piff et al., 2012). Reflecting adaptive logic, sociocultural theories of social class posit an inverse relationship between people’s material resources (e.g., income and education) and their levels of social support (Batruch et al., 2025; Carey and Markus, 2017; Fiske and Markus, 2012; Stephens et al., 2014a). Because working-class contexts afford relatively few material resources, people within such milieus tend to maintain enduring and supportive social ties seen as essential to navigating life’s challenges. More affluent middle- and upper-class contexts, in contrast, enable individuals to leverage financial resources in meeting life’s challenges without necessitating the involvement of close others.

The present work addresses two major challenges to research on social class. First, some researchers question the present-day relevance of social class itself—arguing instead that “gradational” indices (e.g., markers of socioeconomic status or occupational prestige) suffice to capture individuals’ standing within society (e.g., Blau and Duncan, 1978; Kingston, 2000; Treiman, 1977). As Grusky and Weeden (2006) note, rejoinders to the gradational critique have typically appealed to theoretical tradition rather than data. We thus sought to demonstrate the unique and irreducible existence of distinct social-class cultures—or groups of individuals who share common material affordances and behaviors that coordinate social life in a given economic milieu. Second, recent replication efforts have called into question the reproducibility of many social-class effects on human psychological functioning—including the inverse relationship between class and social support (Batruch et al., 2025). We suggest that by carefully decomposing class phenomena into individual-level and group-level processes, we can faithfully represent sociocultural theories of class while also shedding light on these replication failures.

### Culture cycles and the multilevel nature of class processes

**Figure 1:**
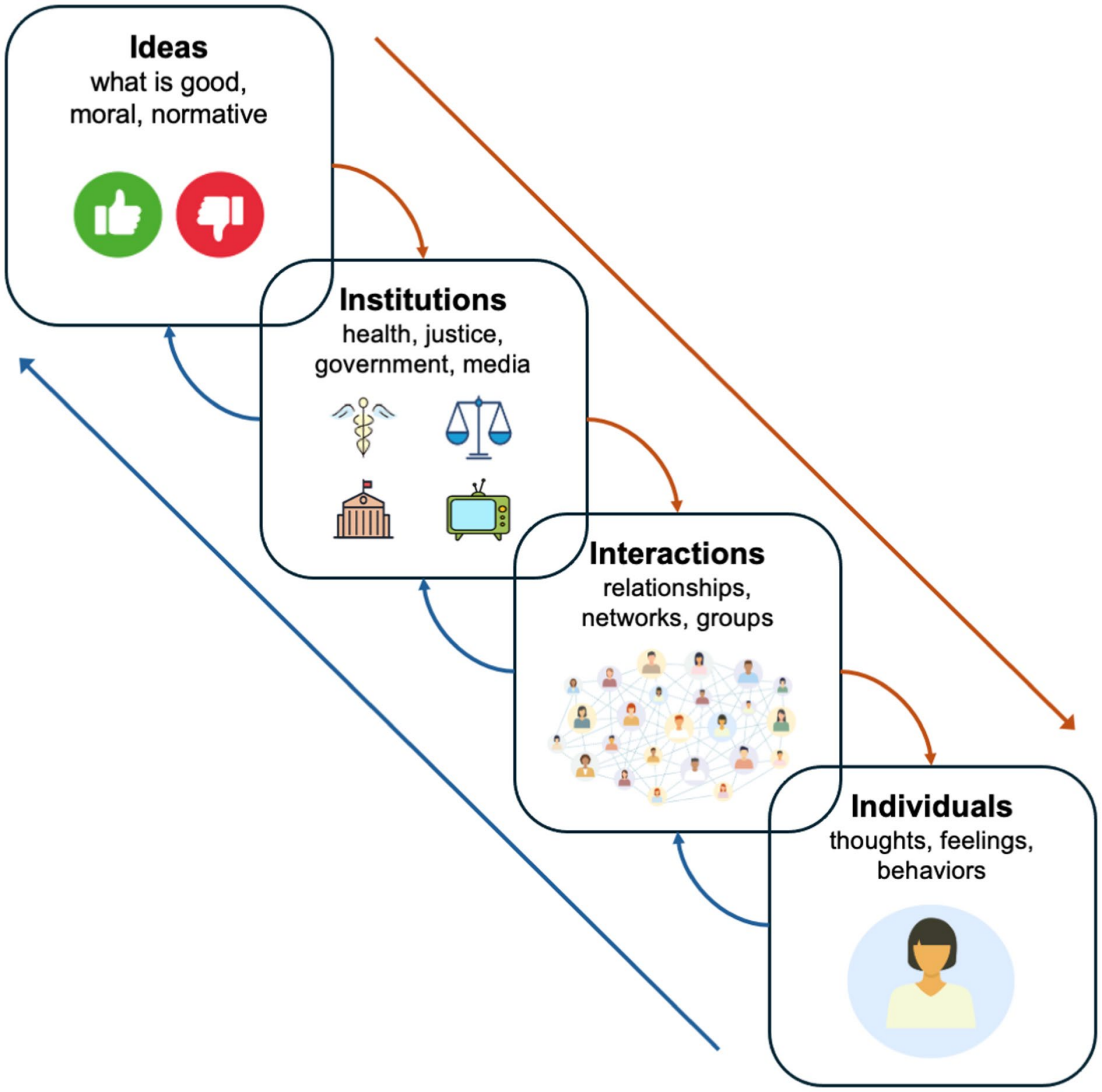
The culture cycle. Orange arrows represent “top-down” effects of the cultural context; blue arrows depict “bottom-up” effects of individual agency. Straight arrows stand in for all other links (e.g., ideas to interactions, individuals to institutions) not otherwise depicted. Adapted from Hamedani and Markus (2019).

Cultural-psychological processes span between individuals and their cultural contexts as person and culture “make each other up” (p. 420; Markus and Kitayama, 2010; see also Shweder, 2003). Such mutually constitutive links between persons and cultures can be understood in terms of the *culture cycle* (Figure 1; Hamedani and Markus, 2019; Stephens et al., 2014b). In this framework, people shape their culture from the “bottom up” while simultaneously being shaped by their culture from the “top down.” In the bottom-up portion of the cycle, people’s thoughts, feelings, and preferences affect their interactions with the environments, institutions, and social representations that prevail within a culture. In the top-down portion of the cycle, broader cultural contexts—with their associated institutions, interaction patterns, and ideas—shape individual psychology. This part of the cycle conveys that many cultural phenomena reflect contextual or group-level effects that are not wholly reducible to individual agency. The present work analyzes a key cultural-psychological claim regarding social class through the multilevel lens of the culture cycle.

A core tenet of the cultural-psychological approach to class is that people’s use of interpersonal resources in everyday life tends to vary inversely with the availability of material resources (Carey and Markus, 2017; Fiske and Markus, 2012; Stephens et al., 2014a). Many of life’s challenges can be addressed either with the help of close others or the purchase of services. Whereas material scarcity can necessitate interpersonal solutions to difficult circumstances, relative affluence renders social solutions optional. For example, a working-class parent who falls ill might need to call upon friends or relatives to care for their child, while a middle-class parent has the option of hiring a babysitter. Crucially, the hypothesized connection between material resources and social support can be interpreted in terms of the bottom-up (i.e., individual-level) *or* the top-down (group- or context-level) phase of the culture culture cycle.

On the one hand, social class differences in social support might reduce entirely to *individual affordances*. Relatively impoverished individuals within either class context will tend to rely on close others more than their wealthier within-class peers, with group-level differences in behavior reflecting the fact that working-class individuals tend to be poorer than their middle-class counterparts. In this way, cultural differences are individual differences “writ large” (Na et al., 2020, p. 908; Smith and Bond, 1999), with between-class variation in social support reflecting the aggregate of individuals’ behaviors within their respective socioeconomic contexts. Given enough time and contextual stability, such behavioral tendencies may “accrete” in the form of culturally prescribed interaction patterns, institutions, norms, and values, thus exemplifying the manner in which culture can be shaped through the exercise of individual agency and decision-making. If this is the case, analytically the hypothesized class difference in social support is equally (in fact, more) recoverable as a continuous association between material resources and support-seeking behavior than as a mean difference between class groups.

On the other hand, the association between material resources and social support could reflect the top-down portion of the culture cycle, or *cultural affordances*. This view affirms that human behavior is shaped not only by people’s appraisals of their immediate circumstances but also by individuals’ broader ecologies—including their physical environments, institutions, social networks and groups, as well as the norms, values, and self-construals that predominate within their culture (Carey and Markus, 2017; Markus and Kitayama, 1991). As such, working- and middle-class cultures foster behaviors and cognitions that adapt individuals to conditions of relative scarcity or abundance. This group-level interpretation implies an inverse relationship between material means and social support *between social-class groups*, with average differences existing independently of any within-class relationships between material resources and social support. Analytically, this kind of association is not necessarily recoverable when tested solely at the individual level (Na et al., 2010).

Our argument echoes that of sociologists and cultural psychologists who emphasize that some social phenomena are irreducibly contextual in nature. In sociology, this fact is often understood in terms of “neighborhood effects,” in which individual outcomes vary independently as a function of person- and group-level characteristics (Diez Roux, 2001). In one famous example, Gelman et al. (2005) used the logic of neighborhood effects to rebut claims that the Republican Party had, through its emphasis on cultural wedge issues like gun rights and sexual morality, become the party of low-income Americans (Frank, 2005). The authors showed that, although voters in lower-income states were more likely overall to support Republican candidates, poorer individuals were *less* likely to vote Republican than their richer within-state peers. Similarly, cultural psychologists have shown that measures of social orientation and cognitive style are correlated as theory would suggest when comparing countries’ mean scores—but uncorrelated at the individual level within any given country (Na et al., 2010). Findings such as these demonstrate the separability of individual and collective phenomena, as well as the importance of appropriately modeling cultural effects at each level.

### Distinguishing individual- and group-level associations between material resources and social support

As exemplified by the culture cycle, cultural-psychological theorizing emphasizes the bidirectional, multilevel processes through which culture and individual psychology shape one another (Hamedani and Markus, 2019; Markus and Kitayama, 2010; Stephens et al., 2014b). Nevertheless, we know of few empirical attempts to parse the degree to which specific cultural phenomena are more reliably detected at the individual versus group level (but see Na et al., 2010; Na et al., 2020)^1^. Thus, the present research sought to disentangle the extent to which associations between material resources and social support better reflect the top-down influence of cultural affordances versus the bottom-up influence of individual affordances.

A pervasive, yet underappreciated, ambiguity arises when individual-level markers of social class (e.g., financial assets or educational attainment) are used to predict outcomes of interest (a practice common among class researchers, including the present authors). Provided that one’s sample contains respondents from multiple class groups, any observed relationship will inevitably conflate the effects of person- and group-level variation in the class proxy.

To illustrate, previous research has found that educational attainment predicts the amount of time people spend looking at human beings in still images, such that those without a four-year degree devote more attention to others than do those with a four-year degree (Dietze and Knowles, 2016, Studies 2a and 2b). Although the class proxy (educational attainment) robustly predicted patterns of visual attention, the data provide no information concerning the individual- versus group-level nature of the education–attention relationship. Indeed, the observed association may reveal something about individuals (e.g., when a person gets a college degree they start looking less at others) or something about the class context (e.g., contexts that afford few opportunities for higher education foster a tendency to look more at other people). To infer from these data that a college education reduces chronic attention to others risks an “ecological fallacy” wherein group- or context-level effects are mistaken for individual-level effects (Robinson, 1950). The reverse inference, that context drives attentional tendencies, risks an “atomistic fallacy” in which individual-level effects are misattributed to higher-level processes (Diez-Roux, 1998).

Conflating group- and individual-level effects can also obscure phenomena that occur at one of these levels but not the other. Na et al. (2010) demonstrate this via mathematical simulation, showing that large, group-level differences can be accompanied by small, even negligible individual-level associations between two variables. Thus, individual-level analytic techniques risk overlooking cultural phenomena that occur exclusively or primarily at the group level. For instance, a recent high-powered replication effort revealed small *positive* individual-level associations between social support and class proxies in pooled samples from multiple countries ( β _education_ = 0.07; β _income_ = 0.05; Batruch et al., 2025). Such findings are easily misconstrued as disconfirming the theorized inverse relationship between class and social support when in fact this relationship might be detectable between, not within, social-class groups. In other words, previous work may have focused on testing the bottom-up portion of the cultural cycle while leaving top-down cultural processes unassessed.

Another common feature of research on social class is the assumption of linear relationships between class indicators and outcomes of interest (Stephens et al., 2024). This approach overlooks the unique psychological effects of extreme deprivation, which may promote isolation rather than social support. Interviews with women on welfare reveal a form of “hard individualism” defined by self-reliance and a lack of trust in others (Kusserow, 1999; Steele and Sherman, 1999). Compared to working-class women, women in poverty report fewer connections to family and friends and experience greater challenges in close relationships (Stephens et al., 2014a). Hence, fewer material resources are not always accompanied by more plentiful social support. Conflating within- and between-group processes hampers analysts’ ability to test such nonlinear hypotheses. Indeed, if nonlinear patterns in the relationship between materials means and social support occur between social-class contexts, even nonlinear models estimated at the individual level (e.g., polynomial regression or generalized additive models) may fail to recover the relevant effect due their assumptions that all individuals are drawn from a single population characterized by a continuous functional form. In contrast, our theoretical framework predicts the presence of qualitatively distinct subgroups, which are not well captured by nonlinear functions.

To address these limitations, we sought an analytic method capable of modeling effects at nested levels (e.g., individuals within groups) and identifying nonlinear group-level relationships. Multilevel modeling (MLM) is a tempting choice, as it enables the analyst to regress outcomes of interest simultaneously on characteristics of the person and the context. In the present work, our data lacked the independently defined nesting units required by MLM. Indeed, the constructs whose group and individual relationships we wish to model—material resources and social support—are precisely the ones that might underpin the higher-level units. Because of these limitations, we sought a different analytic approach.

### The present research

As an alternative to MLM, we used latent profile analysis (LPA) to model between- and within-group relationships between material resources and social support. A form of mixture modeling, LPA tests the likelihood that a variable of interest samples two or more distributions characterized by different means (Oberski, 2016). Different combinations of means across multiple indicator variables reveal distinct subgroups in the population (e.g., a subgroup marked by high means on one variable, low means on a second variable, and a middling mean on a third variable). Like other techniques used to identify groups of similar individuals, such as latent class analysis (LCA) or cluster analysis, LPA is a “person-centered” analytic approach (Pastor et al., 2007). Unlike other approaches, however, LPA allows researchers to assess between-profile differences in respondents’ indicator means while simultaneously modeling associations between indicator variables within the subgroups that emerge from the analysis.

LPA is well-suited to teasing apart between- and within-group phenomena when the groups in question are “hidden”—that is, unknown independently of one’s analytic variables (Oberski, 2016). As such, we assess relationships between material resources and social support between and within potential class contexts identifiable only through reference to these same variables. Informed by cultural-psychological theorizing (e.g., Stephens et al., 2024), we expected that individuals would cluster into distinct groups delineated by mean levels of material resources and social support. However, such clustering is by no means guaranteed. Whereas other techniques, such as MLM, presuppose the existence of meaningful higher-level groupings, LPA renders the existence of subgroups a strictly empirical question. Thus, before attempting to document contextual and individual-level relationships between material resources and social support, we were first required to demonstrate that groups emerge in the first place. Provided that clusters do emerge, we can then evaluate whether relationships between material resources and social support occur more reliably between groups (suggesting a group-level process) or within groups (suggesting an individual-level process).

We subjected two publicly available datasets (*N_total_* = 3,347) to LPA. Consistent with theory that casts social class as a cultural context (e.g., Carey and Markus, 2017; Fiske and Markus, 2012; Stephens et al., 2024), we expected to identify at least two class-cultural groups displaying an inverse relationship between material resources and social support: (1) a working-class group exhibiting relatively low levels of material resources but high levels of social support and (2) a middle- or upper-class group displaying relatively high levels of material capital (i.e., income and education) but low levels of social support. Counter to common linear assumptions—but consistent with sociological and cultural-psychological studies of poverty (Kusserow, 1999; Steele and Sherman, 1999; Stephens et al., 2014a)—we expected to observe a third group combining extreme material deprivation with relatively low levels of social support. Clusters consistent with these expectations emerged in Study 1 and were replicated in Study 2’s preregistered analyses.

Having identified social-class groupings consistent with sociocultural models of social class (e.g., Stephens et al., 2024; Steele and Sherman, 1999), we next tested whether these group-level patterns were detectable using purely individual-level analyses. To this end, we modeled linear and nonlinear relationships between material resources and social support in each study’s full sample. Findings in which the observed group-level relationships prove unrecoverable at the individual level would help explain failures to replicate key social-class effects (Batruch et al., 2025). Specifically, it may be that common approaches look for class effects in “all the wrong places” by modeling hypothesized phenomena at the individual-level rather than the group-level.

## Materials and methods

### Transparency and openness

All datasets, data exclusions, and measures used in the research are described below. Prepared data files, materials, analysis code, and Supplementary materials for both studies are posted on the project’s Open Science Framework (OSF) page. The methods and analyses used in Study 2 were pre-registered at AsPredicted. Although our Study 2 preregistration includes analyses of physical and mental health outcomes, we focus here on social-class differences in social support seeking and availability.

### Samples

#### Study 1

The Study 1 data were retrieved from the General Social Survey (GSS; Davern et al., 1972) website. The GSS is an ongoing series of nationally representative surveys in the United States detailing trends in social opinions, attitudes, and behaviors. Because 2018 was the most recent GSS year containing adequate measures of income, educational attainment, and our preferred measure of social support, we restricted our analyses to 2018 respondents. Additionally, we limited our analyses to respondents who had viable responses to all three variables of interest (i.e., income and/or wealth, educational attainment, and social support). Respondents under 25 were excluded, thus omitting most current college and university students.^2^ Ages ranged from 25–89 (*M* = 50.78, *SD* = 16.34). 45% of the sample had a college degree or advanced degree (e.g., MBA, PhD, JD, or MD) and respondents’ mean income was $35,395 (*SD* = $32,096). The majority of the sample was White (73% White, 16% Black, 11% Other) and female (52%). After exclusions, our final analysis sample consisted of 939 respondents.

#### Study 2

The Study 2 sample was obtained through the National Social Life, Health, and Aging Project (NSHAP; Waite et al., 2017)—a nationally representative study of relationships and healthy aging. We restricted our sample to those respondents with viable responses to each of our three indicator variables in Wave 3 (collected 2015–2016) and the Covid-19 substudy (collected 2020–2021). Ages ranged from 49–94 (*M* = 67.17, *SD* = 9.74). 34% of the sample had a college degree or advanced degree, respondents’ mean income bracket was $50,000–$99,999, and respondents’ mean asset bracket was $100,000–$499,999. Participants were mostly female (56% female) and mostly White (73% White, 14% Black, 9% Hispanic, 3% Other). Our final analysis sample consisted of 2,408 participants.

### Measures

#### Access to material resources

In Study 1, we used respondents’ household income and educational attainment (1 = four-year degree or higher, 0 = no four-year degree) as our primary measures of access to material resources. In Study 2, household income was replaced with a composite of income and financial assets—educational attainment was again used as an additional proxy of material resources. Assets are an important component of material capital (and thus people’s class positioning) as they represent wealth that can be marshaled in times of crisis (Oliver and Shapiro, 2006). Similarly, a college degree is a form of “human” capital that can be converted over the lifespan into financial earnings and wealth. In this sense, college education is an indicator of access to material resources in the future (Becker, 2009; Schultz, 1961).

#### Social support

##### Study 1

In Study 1, social support was measured using ten GSS variables pertaining to support-seeking behavior (Table 1). These items capture whom respondents could turn for help with various everyday tasks and larger life problems. “Everyday task” items were dichotomized such that “*No one*” equals 0 and responses indicating a social tie (e.g., friends, family, acquaintances) equal 1; “life problem” items were dichotomized such that organizations/private companies options and “*No person or organization*” were coded as ‘0’ and responses indicating social ties were coded as 1. Thus, a value of 1 in this coding scheme indicates that the respondent had a person in their social network that they could turn to for help with a particular everyday task or life problem. The items were then averaged to form an index of respondents’ use of social relationships and interpersonal networks to cope with life’s difficulties.

**Table 1:** General Social Survey (GSS) items measuring support-seeking behavior in Study 1 (*α* = 0.56).

GSS variable name	Question
Everyday tasks	
	*Who would you turn to first to…*
HLPHOME	help you with a household or a garden job that you cannot do yourself?
HLPSICK	help you around your home if you were sick and had to stay in bed for a few days?
HLPDOWN	be there for you if you felt a bit down or depressed and wanted to talk about it?
HLPADVCE	give you advice about family problems?
HLPSOCOC	enjoy a pleasant social occasion with?
Life problems	
	*Who would you turn to first to…*
HLPLOAN	help you if you needed to borrow a large sum of money?
HLPJOB	help you if you needed to find a job?
HLPPAPER	help you with administrative problems or official paperwork?
HLPRESDE	help you if you needed to find a place to live?
HLPSICKR	look after you if you were seriously ill?

Everyday task response options: 1 = *Family members or close friends*, 2 = *More distant family members*, 3 = *Close friend*, 4 = *Neighbor*, 5 = *Someone I work work*, 6 = *Someone else,* 7 = *No one*. Options 1, 2, 3, and 4 were coded as 1 for analyses, all other responses were coded as 0. Life problem response options: 1 = *Family members or close friends*, 2 = *Other persons*, 3 = *Private companies*, 4 = *Public services*, 5 = *Nonprofit or religious organizations*, 6 = *Other organization,* 7 = *No person or organization*. Options 1 and 2 were coded as 1 for analyses, all other response options were coded as 0.

We highlight here that, because this measure is conceptualized as a count of available ties rather than a set of interchangeable indicators of a single latent construct, internal consistency metrics such as Cronbach’s *α* are not well-suited to this type of measure. However, this operationalization captures the quantity—but not the quality or strength—of social support.

##### Study 2

In Study 2, social support was measured using four NSHAP variables relevant to perceived social support (Table 2). These items assess the degree to which respondents feel they can rely on family members and friends in times of need. Scores were averaged to form a composite reflecting the degree to which respondents rely on their interpersonal relationships for social support.^3^ In this way, Study 2’s measure speaks more to the quality or strength of social support within respondents’ networks.

**Table 2:** National Social Life, Health, and Aging Project (NSHAP) items assessing perceived social support in Study 3 (*α* = 0.97)

NSHAP variable name	Question
FAMRELY2	How often can you rely on members of your family for help if you have a problem?
FAMFEEL	How often do members of your family really understand the way you feel about things?
FAMOPEN2	How often can you open up to members of your family if you need to talk about your worries?
FRRELY2	How often can you rely on your friends for help if you have a problem?
FRFEEL	How often do your friends really understand the way you feel about things?
FROPEN2	How often can you open up to your friends if you need to talk about your worries?

Item options: 0 = *never*; 1 = *hardly ever or rarely*; 2 = *some of the time*; 3 = *often*.

## Results

### Latent profile analysis

Mplus 8.5 software (Muthen and Muthen, 2017) was used to test LPA solutions in our two studies, treating the three variables of interest—income, educational attainment, and social support—as profile indicators. Prior to the LPA, income and social support were scaled from 0 to 1, with 0 representing the lowest observed score and 1 representing the highest observed score. Educational attainment was coded such that 0 = no four-year college degree and 1 = four-year college degree. Restricted maximum likelihood estimation was specified. Many sets of random starting values (10,000) were tested for each model, increasing our confidence that the estimation algorithm found global (rather than local) log-likelihood maxima and thus the most probable latent groups (Lewis-Beck et al., 2004).

In line with Pastor et al. (2007), we tested models specifying different numbers of profiles and alternative variance–covariance structures for the continuous profile indicators (i.e., income and social support). Our models specified from 1 to 10 profiles. For each number of profiles, we tested a simple model constraining the variance of the continuous indicators to equality across profiles and constraining the covariance between these indicators to zero (A models). More complex sets of models were then fitted: B models, which retained the A models’ single variance estimates but freed the continuous indicators to covary equally across profiles; and C models, which retained the A models’ zero-covariance constraint but freed the continuous indicators’ variances to differ between profiles. Finally, two progressively more complex models were fitted: D models, which freely estimated variances in each profile and allowed a single covariance across profiles; and E models, which freely estimated both the continuous indicators’ variances and their covariance in each profile.

### Model selection principles

The primary goal of LPA is to identify subgroups within a population on the basis of shared attributes (Spurk et al., 2020). The analyst attempts to identify a well-fitting, theoretically useful, and parsimonious statistical solution—that is, one that accurately reflects contours in the data without “overfitting” them by estimating more model parameters than necessary (Pastor et al., 2007; Spurk et al., 2020; Weller et al., 2020). To this end, researchers estimate a range of models specifying different numbers of profiles and alternative variance–covariance structures for the continuous indicators (Pastor et al., 2007). Choosing an optimal solution involves quantitative, theoretical, and subjective considerations (although the use of rigorous model-selection criteria makes LPA less subjective than other clustering techniques; Pastor et al., 2007, p. 14). As such, it is important that researchers make explicit their rationale for selecting a model so that other researchers can evaluate this choice (Spurk et al., 2020).

In a first cut, candidate models can often be ruled out on the basis of model-estimation issues. A common problem occurs when the estimation algorithm is unable to replicate the log likelihood of the best-fitting solution, even with many sets of random starting values. Nonreplication reduces confidence that the algorithm has identified a global (rather than local) log-likelihood maximum and suggests that the model is poorly defined for the data (Geiser, 2013). Another issue arises when the algorithm resorts to “boundary estimates” (i.e., values at the theoretical minimum or maximum for a profile indicator) in order to avoid a singular information matrix. Boundary estimates can indicate that the model is nonidentified or that too many profiles have been requested (Geiser, 2013). Finally, the estimation algorithm may yield untrustworthy standard errors, or fail completely, due to matrix problems.

Once LPA solutions have been screened for estimation problems, the overall fit of the remaining models may be assessed using an array of indices—most commonly the Bayesian information criterion (BIC), sample-size adjusted BIC (SABIC), and Akaike information criterion (AIC; Spurk et al., 2020). Moreover, the effect of specific parameterizations on fit can be assessed using various model-improvement tests. χ ^2^-difference tests are useful in comparing alternatives that specify different variance–covariance structures for the same number of profiles; conversely, the adjusted Lo–Mendell–Rubin likelihood ratio test (LMR-LRT; Lo, 2001; Pastor et al., 2007) or parametric bootstrapped likelihood ratio test (BLRT; McLaughlin et al., 2003) is used to compare models with different numbers of profiles but the same variance–covariance specification.

Unfortunately, comparison of overall fit and use of model-improvement tests cannot always be relied on to prevent overfitting. As profiles are added, BIC may continually decrease and model-improvement tests (e.g., the BLRT) may never reach nonsignificance, tempting the analyst to extract more profiles than are generalizable, theoretically interpretable, or practically useful (Ferguson et al., 2020; Masyn, 2013). In such cases, one can plot the decrease in model BIC (Masyn, 2013) or increase in log likelihood (Ferguson et al., 2020) as profiles are added, inspecting the trend for an inflection point, or “elbow,” after which the rate of model improvement diminishes (elbow test; Masyn, 2013). Similar to the use of scree tests in selecting factor-analytic solutions, the researcher may opt to reject LPA models beyond this point.

Inspection of fit indices, model-improvement tests, and BIC or log likelihood plots may still leave the analyst with an overly complex model. Thus, researchers should carefully examine sample statistics associated with each candidate model. A model that produces one or more extremely small profiles may be rejected, with researchers suggesting cutoffs of 3% or 1% of the overall sample (Spurk et al., 2020) or fewer than 25 cases (Lubke and Neale, 2006). Candidate LPA models should also be examined for evidence of misspecification (Pastor et al., 2007). For instance, if a model fixes the covariances of continuous indicators to zero, but the outputted profiles contain significant correlations, then the model may be misspecified. Likewise, if a model constrains the variance of an indicator to equality across profiles, and yet the indicator’s variance differs significantly between profiles, then the model’s parameterization is called into question.

### Selection of preferred solutions

#### Study 1

Twenty models were immediately rejected due to estimation issues—7 models (5C, 5E, 6C, 6D, 9B, 10A, and 10B) that could not be replicated with 10,000 sets of random starting values (suggesting poor model identification; (Geiser, 2013) p. 241), 5 models (7C, 7E, 8C, 9C, and 10E) that contained boundary estimates (suggesting the extraction of too many profiles; (Geiser, 2013)), and eight models (6E, 7D, 8D, 8E, 9D, 9E, 10C, and 10D) that yielded both errors.

Inspection of sample statistics for the remaining models revealed serious problems with most of those specifying more than three profiles. Specifically, 32 solutions (4A–D, 5A, 5B, and 5E, and all those specifying more than five profiles) yielded very small (or even empty) profiles (see Supplementary Tables S1–S5). We therefore rejected these models despite their comparatively low BIC values. Moreover, examination of sample statistics for solution 5B revealed a potential misspecification of the variance–covariance structure—such that the model constrained indicator variances to equality across profiles even though the outputted profiles exhibited many significant pairwise variance differences. We thus rejected model 5B.

Of the remaining models, we focused on three with the lowest BIC: 3D, 3E, and 4E. Plotting the improvement in BIC as profiles were added to the E models, we observed an inflection point at three profiles—suggesting, per the elbow test, that 4E is an overfitted solution. We thus rejected this model. Finally, because a χ ^2^-difference test showed that model 3E fit the data better than model 3D, we rejected 3D and retained 3E as our preferred solution.

#### Study 2

As in Study 1, candidate LPA models were compared using the Bayesian information criterion (BIC), χ 2-difference tests, the BLRT (McLachlan and Peel, 2000), the elbow test (Ferguson et al., 2020; Masyn, 2013), and examination of sample statistics (Pastor et al., 2007).

Twenty-seven models were immediately rejected due to estimation issues. Specifically, five solutions (4E, 5E, 7B, 8B, 10B) could not be replicated even with 10,000 random starts (suggesting poor fit to the data; Geiser, 2013, p. 244), five solutions (9A, 9D, 10C-E) contained boundary estimates (suggesting the extraction of too many profiles; Geiser, 2013, p. 241), two solutions (6C, 7C) yielded derivative matrix errors suggesting model nonidentification, and 11 models (5C-D, 6D-E, 7A, 7D, 8A, 8C-D, 9B-C, 10A) displayed two or more of these problems.

Of the remaining solutions, models 3D–E and 4C–D had the lowest (best) BIC values. Because model 4C had a better BIC than models 3D–E—and model 4D did not provide a significant improvement over 4C—we retained 4C as our preferred solution.

### Interpreting the emergent profiles

**Figure 2:**
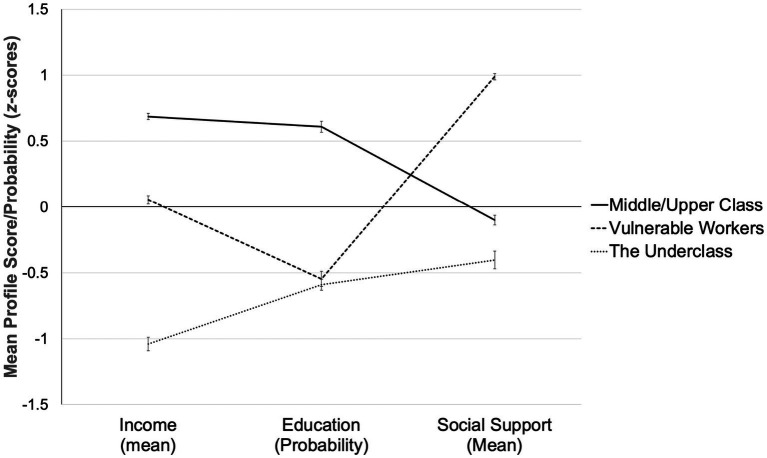
Three social class profiles found in Study 1.

**Figure 3:**
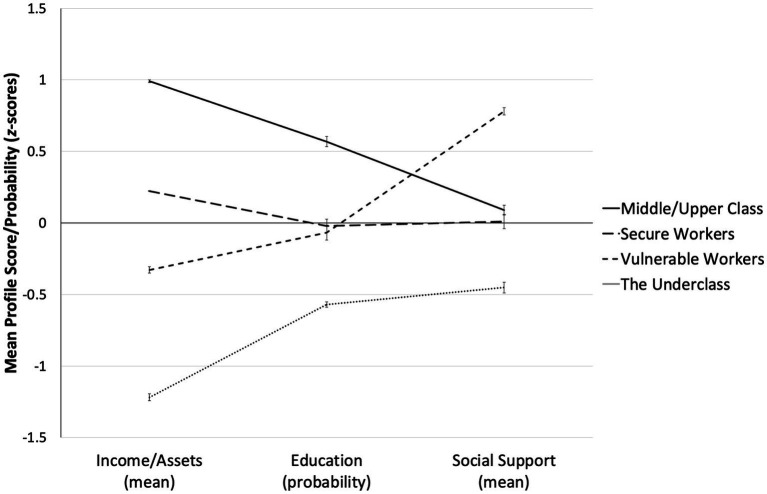
Four social class profiles found in Study 2.

Across our two studies, we find that respondents cluster into profiles marked by different mean levels of access to material resources and mean levels of social support. For a visualization of each group’s profile means, see Figures 2, 3. Respondents in both Study 1 (80% mean classification probability) and Study 2 (78% mean classification probability) were assigned to their respective latent profiles with high certainty (see Supplementary Tables S14, S16 for the mean probability of profile membership in each latent profile). Before examining the profiles” relative levels of social support, we first interpret the profiles in light of their distinct material contexts. In doing so, we sought to give these profiles social-class labels that researchers from a broad range of theoretical perspectives would find appropriate.

Three profiles emerged in Study 1, while four groups were extracted in Study 2. The labels selected are by no means diagnostic, but instead are heuristic labels chosen based on the profiles’ access to material resources, theory from cultural psychology that treats the possession of a four-year college degree as a marker of middle-class status (Stephens et al., 2024), and additional demographic information (e.g., common occupations, class self-identification) available in the datasets. For a summary of the demographic of the profiles, see Tables 3, 4.^4^

**Table 3:** Prevalence and predicted characteristics of class groups found in Study 1.

Profile characteristics	The underclass	Vulnerable workers	Middle/Upper class
*n* (% of sample)	310 (33%)	173 (18%)	456 (49%)
Age	52.34	45.53	51.37
% female	56%	53%	48%
% White	62%	72%	82%
Probability has degree	0.18	0.26	0.72
Mean income	$5,190	$15,222	$65,583
Mean social support	0.75	0.96	0.79
Probability own home	0.37	0.60	0.88
Probability unemployed in last 10 years	0.46	0.32	0.26
Most common occupations	Production, transportation, and material moving	Service occupations	Management, business, and financial
Preferred social class label	“Working class”	“Working class”	“Middle class”
Mean neighborhood income^1^	$57,778	$60,600	$99,170
Neighborhood proportion of individuals with a college-degree	23%	20%	42%

^1^Neighborhood income and education estimates were derived from the American Community Survey (5-year estimates). These data were merged with respondent data using census-tract identifiers present in the GSS database for all respondents who had a valid tract identifier (*n* = 720).

**Table 4:** Prevalence and predicted characteristics of class groups found in Study 2.

Profile characteristics	The underclass	Vulnerable workers	Secure workers	Middle/Upper class
*n* (% of sample)	797 (33%)	316 (13%)	445 (18%)	850 (35%)
Age	67.04	71.39	66.27	65.44
% Female	61%	69%	54%	47%
% White	56%	77%	76%	86%
Probability has degree	0.09	0.34	0.32	0.58
Income	< $25,000	$25,000–$49,999	$50,000–$99,999	$100 k or greater
Assets	$10,000–$49,999	$100,000–$499,999	$100,000–$499,999	$500 k or greater
Mean social support	0.66	0.85	0.72	0.75

#### Middle/upper class

In both studies, we observe a group characterized by relatively abundant material capital and the possession of a college degree; we termed this group the “middle/upper class.” Demographic information available in Study 1 supports this classification: Respondents in the middle/upper-class cluster are relatively likely to own a home, to have avoided unemployment, to work in the management, business, or financial sectors, to self-identity as “middle class,” and reside in relatively affluent neighborhoods (see Table 3). We therefore expect that researchers from a range of perspectives would regard this cluster as an amalgam of middle, upper-middle, and upper class individuals.

#### Secure workers

Another group emerged only in Study 2. This cluster tends to have high levels of material capital but *lack* a college degree. Due to their lack of a college or university education, we regard these respondents as members of the working class. At the same time, these individuals enjoy substantial income and assets, likely providing some protection from unexpected financial shocks. We therefore termed this group the “secure workers.”

#### Vulnerable workers

A third group emerged in both studies. This profile was marked by the lack of a college degree and modest levels of material capital (i.e., income and assets). These respondents tend to work in service occupations, display the second highest rates of recent unemployment of any profile, and live in relatively less affluent neighborhoods. About half of those in this group own their own homes—substantially fewer than secure workers or the middle/upper class. Like secure works, this profile bears hallmarks of the working class. However, their relative material precarity leads us to label them “vulnerable workers,” as they are likely one financial shock away from falling out of the working class altogether.

#### The underclass

A fourth and final group emerged in both studies. Members of this cluster have very low incomes and few assets, are highly unlikely to hold a college degree, experience the highest levels of unemployment of any profile, rarely own a home, and live in less affluent neighborhoods. Given this pattern of material deprivation, this group likely experiences poverty, stigmatization, and exclusion from mainstream society (McLaughlin et al., 2003). While members of this group prefer to self-identify as “working class,” they are also the most likely of any profile to call themselves “poor.” We therefore termed this cluster “the underclass.”

### Are class group level differences detectable at the individual level?

Our LPAs revealed clear and consistent group-level associations between material resources and social support in Studies 1 and 2—with *vulnerable workers* reporting more social support than the *middle/upper class*, and the *underclass* reporting the least social support of any group. We next sought to ascertain whether these material–social relationships were detectable using analytic approaches that ignore group-level effects. In the studies’ full samples, we (1) examined the bivariate correlations between material resources and social support, and (2) estimated general additive models (GAMs) to flexibly detect nonlinear material–social associations.

We first computed the full-sample correlations between material resources and social support in each of our studies. In Study 1, social support (i.e., supportive relationships) was uncorrelated with household income (*r* = −0.02; *d* = −0.03, *p* = 0.632) and education (*r* = −0.02; *d* = −0.03, *p* = 0.644). In Study 2, however, social support (i.e., perceived support) was *positively* correlated with the income/asset composite (*r* = 0.13; *d* = 0.27, *p* < 0.001) and education (*r* = 0.10; *d* = 0.21, *p* < 0.001). For a comparison of the effect sizes detected by our group-level analyses vs. linear associations at the individual level, see Table 5. The failure to recover the observed group-level pattern using simple correlations is perhaps not surprising given the nonlinearity of the material–social associations in our studies. We therefore proceeded to estimate GAMs, which are better suited to flexibly detecting nonlinear patterns.^5^

**Table 5:** Magnitude and direction (Cohen’s d) of associations between material resources and social support at the individual and group levels (Studies 1–2).

	Full Sample	Within-Profile				Group difference in social support (VW vs. MU)
		UC	VW	SW	MU	
Study 1 (*N* = 939)	*−0.03 | −0.03*	−0.97 | −0.16	*0.13 |* 0.47		0.22 | 0.29	−1.60
Study 2 (*N* = 2,408)	0.13 | 0.11	−0.17 | *−0.04*	−0.16 *|* −0.26	*−0.05 |* 0.13	0.08 | *0.04*	−0.82

Income and education associations are located on the left and right sides of the | symbol. All effect sizes are significant (*p* < 0.05) unless indicated with italics. UC, the underclass; VW, vulnerable workers; SW, secure workers; MU, middle/upper class.

Given that education was dichotomous, and therefore unable to enter into nonlinear relationships, we only ran GAMs regressing social support on personal income (Study 1) and the income/asset composite (Study 2). The GAMs included a smooth term for income or income/assets and used a Gaussian distribution with an identity link function, estimated using restricted maximum likelihood (REML). In Study 1, the smooth term for income was nonsignificant, *edf* = 1.00, *F*(1.00, 1.00) = 0.23, *p* = 0.63, yielding no evidence of a nonlinear relationship between income and social support.

In Study 2, the model revealed a positive nonlinear relationship between income/household assets and social support, *edf* = 1.93, *F*(2.39, 1928) = 15.38, *p* < 0.001, such that social support increased more steeply at lower values of income/assets before plateauing at higher values. However, this nonlinear effect clearly fails to recover the pattern observed at the group level—in which extreme material deprivation and relative affluence were associated with the lowest levels of social support. For a depiction of GAM results from Studies 1 and 2, see Supplementary Figure S4.

**Figure 4:**
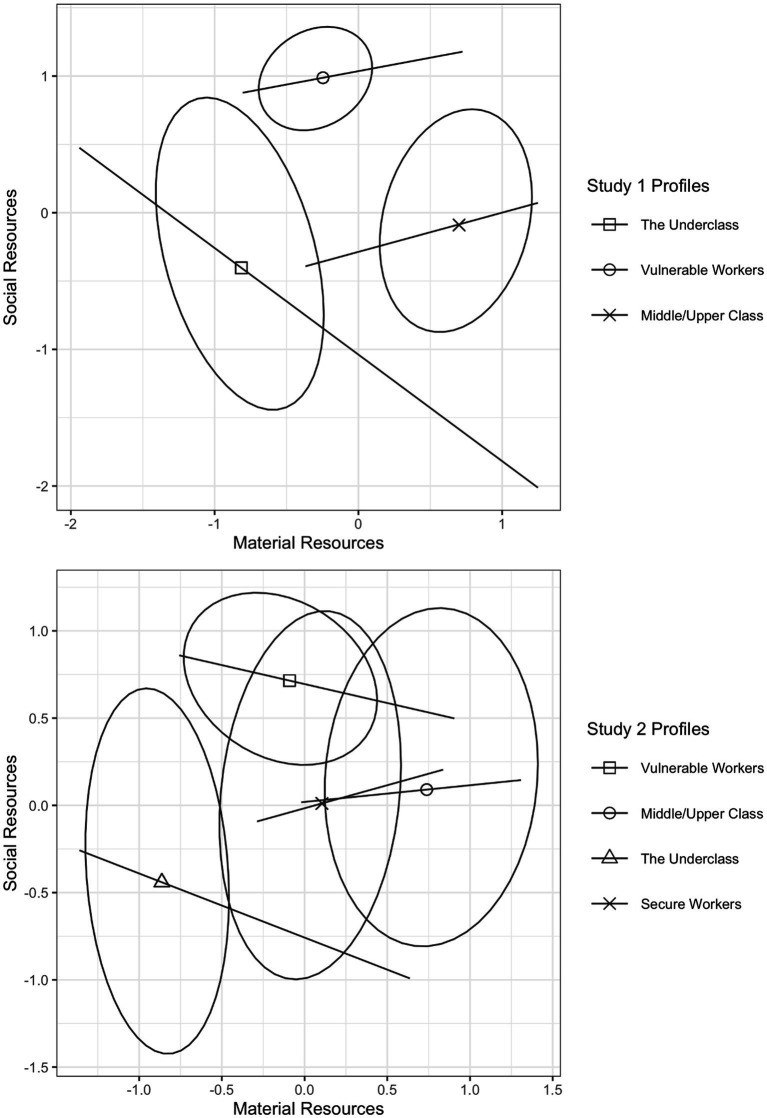
Social-class profile means and within-profile associations between material resources and social support in studies 1–2.

To visualize the split between group-level and individual-level effects in our data, Figure 4 provides visualizations of the distinct between- and within-class relationships between material resources and social support in Studies 1 and 2. Each emergent social-class profile is positioned according to its mean level of material resources on the *x*-axis and mean social support on the *y*-axis, with income and education collapsed into a single indicator for ease of depiction. An ellipse circumscribes 50% of respondents in the class. Overlaid on each ellipse, and passing through the profile mean, is a correlation line representing the within-class relationship between material resources and social support. As can be seen, within-class relationships are generally weak and inconsistent despite large and consistent differences in material resources and social support between the vulnerable worker and the middle/upper class groups.

## Discussion

Social classes are positionings within an economic hierarchy that afford different levels of access to material resources and social support (Piff et al., 2012). Shaped by divergent resource affordances, working- and middle-class cultural contexts promote unique ways of being in response to the resources most abundant within them. Working-class contexts, in which material resources are relatively scarce, tend to foster interdependence as individuals within such contexts emphasize the importance of close, supportive social ties that can be leveraged to navigate contexts defined by limited material resources and greater constraints (Carey and Markus, 2017; Fiske and Markus, 2012). More affluent middle- and upper-class contexts, in contrast, tend to foster independence, autonomy, and self-direction—values compatible with the use of material means to cope with life’s challenges. Nonetheless, typical approaches to studying social class leave an important question unaddressed: Do class-cultural differences in social support reveal the “fingerprint” of culture on individuals’ values—or do they reflect the central tendency of individuals’ personal resource affordances within a class context?

The present research articulates this question in terms of the *culture cycle* (Hamedani and Markus, 2019; Markus and Kitayama, 2010), a framework specifying the “top-down” and “bottom-up” pathways through which culture and mind constitute one another (see Figure 1). The culture cycle frames the manner in which individuals become socialized to the orientations, traits, and behaviors that predominate within a cultural context or group, while also acknowledging that individuals can change their culture by choosing to embrace new norms, values, and selfways. Although both portions of the cycle occur in tandem, we believe that researchers can learn much about specific cultures by examining whether particular phenomena unfold primarily at the level of the individual or the group. As such, the present research sought empirically to distinguish between group-level effects and individual-level effects with respect to the association between material resources and social support. We leveraged latent profile analysis (LPA), a form of mixture modeling, to differentiate relationships between and within emergent class groups.

In two publicly-available datasets (*N_total_* = 3,347), we found that respondents consistently clustered into three social-class categories. We labeled these class categories in a heuristic manner based on their access to material resources, theory from cultural psychology that treats the possession of a four-year college degree as a marker of middle-class status (Stephens et al., 2024), and additional demographic information (e.g., common occupations, class self-identification) available in the datasets. Based on these demographic data, we derived labels which we believe would be deemed appropriate by a broad range of theoretical perspectives. Specifically, we identified: a *middle/upper class* defined by high levels of material resources, a group of *vulnerable workers* characterized by middling levels of material resources, and an *underclass* marked by meager material resources. Comparison of vulnerable workers’ and the middle/upper class’s mean levels of social support reveals a strong group-level effect—such that vulnerable workers displayed much higher levels of interpersonal social support (Studies 1 and 2) than the middle/upper class. This—combined with the fact that members of these groups live in neighborhoods that significantly differ in their overall affluence (Study 1)— suggests that shared material circumstances within social-class groups can explain class differences in the maintenance of social networks rich in supportive ties (Fiske and Markus, 2012; Stephens et al., 2024).

Conversely, we see little evidence for individual-level associations between material resources and social support among either the vulnerable workers or middle/upper class. In fact, we see *positive* (though weak) relationships between material resources and social support among respondents within these class groups. Interestingly, these within-group patterns comport well with models of status hierarchy, such that those highest in material capital tend also to be those highest in social capital (e.g., Bourdieu, 1986). Regardless of their origins, these within-class correlations provide little evidence that individual resource affordances inform the nature of individuals’ social ties. Instead, the presence of negative group-level relationships between material resources and social support, coupled with the absence of such effects at the individual level, provides evidence that the maintenance of supportive social ties is primarily driven by group-level processes that are irreducible to individual-level effects. We further note that these results are robust to different conceptualizations of social support, and that our results are largely the same when social support is operationalized as the quantity of supportive ties (Study 1) and quality of support (Study 2). In the language of the culture cycle, associations between material resources and social support primarily reflect the top-down influence of shared material circumstances.

As a practical matter, the present findings highlight how difficult it can be to detect group-level phenomena without modeling effects at multiple levels. Indeed, in the present studies, a purely individual-level analytic approach yields little evidence for the theorized negative relationship between material resources and social support—a finding in line with recent replication efforts (Batruch et al., 2025). Examining the full-sample linear and nonlinear associations between markers of material resources and social support, we find a null relationship between material resources (income and education) and access to social support (Study 1), and a positive association between material resources (education and a composite of income and assets) and perceived social support in Study 2.

The discrepancy between this lack of evidence for individual-level associations between material resources and social support, and the robust evidence afforded by our LPAs presents no contradiction. Simple correlations between class markers and measures of social support conflate group- and individual-level effects—and therefore obscure phenomena that occur at one of these levels but not the other (Na et al., 2010, 2020). As Na et al. (2010) demonstrate through mathematical simulation, evidence for cultural phenomena occurring primarily at the group-level may be difficult to document using individual-level analyses alone, even when mean differences between groups are very large.

This can be seen by imagining two cultural groups, A and B, that exhibit a large group-level negative relationship between material resources and social support—such that individuals in culture A display a higher mean income level (Cohen’s *d* = 0.8) and lower mean level of social support (Cohen’s *d* = −0.8) than do individuals in culture B. In the absence of any association between income and social support within cultures A or B, the correlation between these variables cannot exceed *r* = −0.14 in the full sample of individuals from both cultures (Na et al., 2010, p. 6194). Thus, individual-level analytical techniques risk overlooking cultural phenomena that occur primarily at the group-level (rather than the individual-level). Our results mirror Na et al.’ (2010) mathematical simulation and emphasize the importance of contending with the multilevel nature of cultural phenomena, because analyzing these phenomena at one level (but not the other) may overlook or discount them entirely.

The present work is rooted in theories that cast social class as a form of culture. According to cultural-psychological analyses of social class, working-class cultural contexts tend to promote interdependent norms, values and selfways in response to economic scarcity, whereas middle- and upper-class contexts afford greater access to material resources and thus foster independent cultural orientations (Stephens et al., 2024). Grounding social-class cultures in resource affordances at the supra-individual level (i.e., context or group) stresses the need to adopt analytic methods capable of distinguishing these higher-order processes from individual-level effects—even when, as in the present research, a single set of analytic variables is used to identify the groups and test relationships between and within them. In this vein, we see LPA as an approach that does justice to the idea of *context in people*—the degree to which everyday contexts and the social realities within them come to shape personal behavior (Adams, 2012; Carey and Markus, 2017)—as well as emphasizing the possibility that interventions targeting class differences at the group-level (rather than the individual-level) may be especially effective.

Although both portions of the culture cycle occur simultaneously (Markus and Kitayama, 2010), we find consistent evidence that the relationship between material resources and social support constitutes a group-level effect that cannot be reduced to individual affordances. We believe this illuminates a critical feature of class contexts in the United States. Specifically, class inequality in the United States is relatively “sticky”—characterized by low economic mobility (Chancel et al., 2022; OECD, 2010), high geographic segregation as a function of income (Grusky et al., 2019), and differential access to educational opportunities (Grusky et al., 2019; Mazumder, 2005; Reeves, 2017). Because inequalities between classes reflect longstanding patterns of stratification, social classes in the United States can be thought of as “mature” cultures whose characteristic norms, values, and selfways have had ample time to develop. In this sense, the top-down influence of class cultures on individuals exerts a greater influence relative to the bottom-up influences of individual affordances regarding social support.

In the present work, we interpret stronger group-level differences as evidence that is more consistent with top-down cultural influences. On the other hand, stronger individual-level associations would be evidence of more prevalent bottom-up influences. Nevertheless, LPA lacks the control necessary to establish causal processes, and using LPA in this manner to proxy the relative strength of either portion of the culture cycle leaves alternative explanations viable in explaining some of the patterns of results we observe. That being said, we believe that rigorously parsing the extent to which cultural phenomena occur at the group- or individual-level may also shed light on the dynamics of cultural formation and change. In societies characterized by high levels of economic mobility, mature class cultures may have yet to form. In such cases, individual-level class phenomena would likely be more pronounced than their group-level counterparts. Individuals in high-mobility contexts would still have to contend with their economic positioning and make adaptive decisions concerning their levels of sociality. But to the extent that this positioning is temporary, social representations regarding the “right” norms, values, and selfways may not fully coalesce. If class mobility were to decrease, such that successive generations are now largely consigned to the same material contexts, the countless individual decisions that constitute the bottom-up portion of the culture cycle might gradually be abstracted into stable cultural ideals. Although more work will be required to model cultural change and formation in terms of these pathways’ relative magnitudes, we believe such a project is theoretically and empirically tractable through other methods, such as agent-based modeling.

Although we regard LPA as a promising means of capturing group-level cultural phenomena, we acknowledge that it will not always be feasible for researchers to analyze their data using this approach. Extracting coherent profiles often requires large samples, which may not always be available to researchers. LPA is also exploratory in so far as the choice of an optimal set of profiles is made by comparing a number of alternative solutions. As others have argued (e.g., Park et al., 2023), these features make it particularly important to (1) validate the retained profile solution using a range of covariates (i.e., examine whether the profiles are associated with other variables in a theoretically-sensible way) and (2) replicate the findings across multiple samples. In the present investigation, for example, we were only able to validate our profiles against external covariates (e.g., neighborhood affluence) in Study 1, because we lacked the geographic identifiers necessary to do so in Study 2. Additionally, our preferred solution in Study 2 produced an additional profile, possibly due to an increased sample size. Nevertheless, our models exhibited high classification quality—suggesting a high probability that individuals “belonged” to the profiles they were assigned to—and our profiles were validated against measures of neighborhood affluence in a sensible manner. Although these points support our claims that these profiles represent strong approximations of shared material contexts, future work should seek to replicate these profiles in other datasets and validate them across a wider range of possible geographic (e.g., neighborhood school quality, healthcare access) and cultural (e.g., shared norms) covariates.

Despite these limitations—and in accordance with prior sociocultural models of class (Carey and Markus, 2017; Stephens et al., 2024)—our data highlight that educational attainment might serve as a reliable proxy for individuals’ class contexts when LPA is unavailable, given that this variable was often the dividing line between the identified working and middle/upper class cultural groups. Moreover, as Study 1 highlights, individuals reliably self-report their class-cultural contexts when prompted to identify with the working or middle classes. Comparing individuals’ LPA classifications with their preferred class labels, we find that if the LPA identified an individual as a member of one of the working class groups, there were roughly 2-to-1 odds that the individual self-identified with the working class. On the other hand, if the LPA identified an individual as a member of the middle/upper-class cultural group, there were almost 2-to-1 odds that the individual self-identified with the middle class. With this in mind, we view educational attainment (dummy-coded as having a four-year degree vs. not), and class-self identification as being two of the more reliable approximations of individuals’ broader class-cultural groups.

Utilizing LPA to study class phenomena can also interrogate linear and monotonic assumptions of social class and its consequences. Class comparisons often assume linear class-based differences in psychology and behavior, such that each gradational increase or decrease in class is expected to yield a congruent difference in some outcome (Stephens et al., 2024). Measuring effects at the group-level, however, tests this assumption. In the present work, for instance, we identified one social class—“the underclass”—which displayed the lowest levels of material resources. The nature of this profile and its effects were unobserved using nonlinear analytic approaches at the individual-level, highlighting another strength of LPA as an analytic tool. Taking a linear approach, past theory might suggest that the individuals with the fewest material resources would behave the most interdependently. However, our results show that the underclass often displayed the lowest levels of social support, suggesting that a linear understanding of class is not wholly accurate. These results corroborate similar findings that people experiencing extreme poverty could experience severe forms of stigmatization and exclusion by mainstream society and have a unique psychological profile (McLaughlin et al., 2003). For example, women on welfare described themselves as less trusting and more independent from others than women from working- and middle-class contexts (Kusserow, 1999; Steele and Sherman, 1999; Stephens et al., 2014b). Adopting a group-based conceptualization of social class helps us make sense of the fact that material deprivation does not always lead to greater social support.

In sum, our results point toward a group-based understanding of class and its consequences. Rather than reflecting “individual differences writ large,” our results highlight that social class is a dynamic cultural context individuals move through and are shaped by in consequential ways. Given that past work often measures class phenomena at the individual level, our results also raise the broader possibility that prior work has *underestimated* effects attributable to class groups. We close by emphasizing that approaches such as LPA provide researchers with the ability to understand the magnitude of cultural effects at multiple levels of analysis.

## Data Availability

Existing datasets are available in a publicly accessible repository: Publicly available datasets were analyzed in this study. This data can be found here: https://osf.io/kxb7c/overviewview_only=62d5c87a817147d6ac4320bcdb80a31c.

## Footnotes

1 Na et al. (2010, 2020) delineate between effects at the individual- and group-level for cross-national cultural contexts. Although the authors make group-level claims about social class, they do so using individual-level analyses (e.g., a dichotomous indicator of educational attainment). This approach, as we explain in the next paragraph, cannot disentangle individual- and group-level class effects.

2 College and university students often do not work and instead receive financial support from their parents, loans, and scholarships, making their class background difficult to measure.

3 We note that our measure of social support diverges from our pre-registration. We had planned to include three additional items in our social-support composite assessing participants’ perceptions of their neighborhood and community (e.g., “Are people in this area willing to help?”). However, we ultimately concluded that these items risked conflating variance in individual and contextual resource affordances (e.g., personal income and neighborhood affluence). Because our research aims required us to carefully distinguish between effects at multiple levels, we excluded these items from our analyses.

4 To obtain estimates of the profile characteristics, we regressed variables of interest simultaneously on the profiles’ posterior probabilities while constraining the intercept term to zero (Pastor et al., 2007). The resulting regression coefficients represent predicted means for each profile, weighted by the accuracy with which individuals can be classified.

5 With general additive models, the data determine the shape of any nonlinear associations. GAMs are therefore more flexible than other nonlinear approaches, such as polynomial regression, that assume nonlinearities of a prespecified form.
