# Supplementary material for: Looking for social class in all the wrong places: differences in social support emerge reliably between—but not within—class contexts
Source: Front Psychol. 2026 Jul 1;17:1688178. doi: 10.3389/fpsyg.2026.1688178 (PMC13368481; doi:10.3389/fpsyg.2026.1688178)

Looking for social class in all the wrong places:

Differences in social support emerge reliably between—but not within—class contexts

SUPPLEMENTAL MATERIALS

Table S1 *Sample Statistics for A Models (Equal Variances and No Covariance) with 2–10 Profiles in Study 1 (N = 939)* **3**

Table S2 Sample *Statistics* for B Models (Equal Variances and Common Covariance) with 2–10 Profiles in Study 1 (*N* = 939) **4**

Table S3 *Sample* Statistics for C Models (Unique Variances and No Covariance) with 2–10 Profiles in Study 1 (*N* = 939) **5**

Table S4 Sample Statistics for D Models (Unique Variances and Common Covariance) with 2–10 Profiles in Study 1 (*N* = 939) **6**

Table S5 Sample Statistics for E Models (Unique Variances and Unique Covariances) with 2–10 Profiles in Study 1 (*N* = 939) **7**

Table S6 Sample Statistics for A Models (Equal Variances and No Covariance) with 2–10 Profiles in Study 2 (*N* = 2,408) **8**

Table S7 Sample Statistics for B Models (Equal Variances and Common Covariance) with 2–10 Profiles in Study 2 (*N* = 2,408) **9**

Table S8 Sample Statistics for C Models (Unique Variances and No Covariance) with 2–10 Profiles in Study 2 (*N* = 2,408) **10**

Table S9 Sample Statistics for D Models (Unique Variances and Common Covariance) with 2–10 Profiles in Study 2 (*N* = 2,408) **11**

Table S10 Sample Statistics for E Models (Unique Variances and Unique Covariances) with 2–10 Profiles in Study 2 (*N* = 2,408) **12**

**Table S11**	*Correlations Between Three Types of Capital in Study 1*	**13**
**Table S12**	*Correlations Between Three Types of Capital in Study 1*	**14**
**Table S13**	*Correlations Between Three Types of Capital in Study 2*	**15**
**Table S14**	*Model Fit (BIC) for Latent Profile Models in Study 1*	**16**
**Table S15**	*Classification Table for Preferred Solution in Study 1*	**17**
**Table S16**	*Model Fit (BIC) for Latent Profile Models in Study 1*	**18**
**Table S17**	*Classification Table for Preferred Solution in Study 1*	**19**
**Table S18**	*Model Fit (BIC) for Latent Profile Models in Study 2*	**20**
**Table S19**	*Classification Table for Preferred Solution in Study 2*	**21**
**Figure S2**	*Log-likelihood and BIC graphs for all models in Study 1 (N = 939)*	**22**
**Figure S3**	*Log-likelihood and BIC graphs for all models in Study 2 (N = 2,408)*	**23**
**Figure S4**	*Generalized Additive Model graphs in Studies 1–2*	**24**

Table S1

Sample Statistics for A Models (Equal Variances and No Covariance) with 2–10 Profiles in Study 1 (N = 939)

	Number of Profiles								
	2	3	4	5	6	7	8	9	10
Estimation problems	No	No	No	No	No	No	No	No	Yes
Profiles containing less than 3% of sample[1]	0	0	1	1	1	2	3	4	4
Profiles containing less than 1% of sample[1]	0	0	0	0	0	0	2	3	3
Profiles containing fewer than 25 cases[1]	0	0	1	1	1	2	3	3	4
Empty profiles	0	0	0	0	0	0	0	0	0
Pairwise variance differences (material capital) / maximum possible[2]	0 / 1	0 / 3	3 / 6	7 / 10	6 / 15	8 / 21	8 / 28	4 / 36	4 / 45
Pairwise variance differences (social capital) / maximum possible[2]	1 / 1	2 / 3	2 / 6	2 / 10	3 / 15	0 / 21	0 / 28	7 / 36	9 / 45
Profiles with significant correlations	0	2	0	0	0	1	1	0	0
Mean correlation strength (weighted)	r = .056, p = .088	r = .087, p = .008	r = .063, p = .056	r = .037, p = .258	r = .05, p = .128	r = .066, p = .044	r = .066, p = .044	r = .091, p = .006	[illegible]= .098, p = .00[illegible]
Correlation min / max	r = -.11 / -.044	r = -.173 / .012	r = -.115 / .097	r = -.032 / .27	r = -.137 / .241	r = -.349 / .087	r = -.349 / .239	r = -.137 / 1	r = -.216 / 1
Pairwise correlation differences / maximum possible	0 / 1	1 / 3	0 / 6	0 / 10	0 / 15	2 / 21	2 / 28	0 / 36	0 / 45
Significance of correlation differences	p = .432	p = .068	p = .667	p = .766	p = .477	p = .229	p = .264	—[3]	—[3]

[1]Possible cutoff for excessively small profiles (Spurk et al., 2020). [2]Indicators of possible variance-covariance misspecification. [3]Could not be computed with *mvtest* in Stata (Stata Corp., 2021) due to small classes.

Table S2

Sample Statistics for B Models (Equal Variances and Common Covariance) with 2–10 Profiles in Study 1 (*N* = 939)

	Number of Profiles								
	2	3	4	5	6	7	8	9	10
Estimation problems	No	No	No	No	No	No	No	Yes	Yes
Profiles containing less than 3% of sample[1]	0	0	1	0	1	2	3	3	4
Profiles containing less than 1% of sample[1]	0	0	0	0	0	0	2	1	3
Profiles containing fewer than 25 cases[1]	0	0	1	0	1	2	3	3	4
Empty profiles	0	0	0	0	0	0	0	0	0
Pairwise variance differences (material capital) / maximum possible[2]	0 / 1	0 / 3	3 / 6	4 / 10	6 / 15	8 / 21	7 / 28	19 / 36	16 / 45
Pairwise variance differences (social capital) / maximum possible[2]	1 / 1	2 / 3	2 / 6	3 / 10	3 / 15	0 / 21	0 / 28	0 / 36	0 / 45
Profiles with significant correlations	0	2	0	2	0	1	1	2	5
Mean correlation strength (weighted)	r = .063, p = .054	r = .098, p = .002	r = .068, p = .036	r = .12, p = 0	r = .069, p = .034	r = .077, p = .018	r = .085, p = .01	r = .133, p = 0	r = .247, p = 0
Mean correlation strength	r = -.124 / -.049	r = -.196 / .004	r = -.126 / .097	r = -.217 / -.024	r = -.156 / .041	r = -.349 / .041	r = -.349 / .239	r = -.447 / .061	r = -.711 / .033
Pairwise correlation differences / maximum possible	0 / 1	2 / 3	0 / 6	0 / 10	0 / 15	0 / 21	1 / 28	0 / 36	1 / 45
Significance of correlation differences	p = .374	p = .041	p = .533	p = .521	p = .725	p = .549	p = .447	p = .921	p = .397

[1]Possible cutoff for excessively small profiles (Spurk et al., 2020). [2]Indicators of possible variance-covariance misspecification.

Table S3

Sample Statistics for C Models (Unique Variances and No Covariance) with 2–10 Profiles in Study 1 (*N* = 939)

	Number of Profiles								
	2	3	4	5	6	7	8	9	10
Estimation problems	No	No	No	Yes	Yes	Yes	Yes	Yes	Yes
Profiles containing less than 3% of sample[1]	0	0	1	0	1	2	3	4	5
Profiles containing less than 1% of sample[1]	0	0	0	0	0	1	2	3	4
Profiles containing fewer than 25 cases[1]	0	0	1	0	1	2	3	4	5
Empty profiles	0	0	0	0	0	1	2	3	4
Pairwise variance differences (material capital) / maximum possible[2]	1 / 1	3 / 3	5 / 6	9 / 10	14 / 15	14 / 21	14 / 28	14 / 36	14 / 45
Pairwise variance differences (social capital) / maximum possible[2]	1 / 1	3 / 3	4 / 6	5 / 10	9 / 15	9 / 21	9 / 28	9 / 36	9 / 45
Profiles with significant correlations	2	1	2	1	0	0	0	0	0
Mean correlation strength (weighted)	r = .172, p = 0	r = .164, p = 0	r = .122, p = 0	r = .051, p = .116	r = .051, p = .118	r = .051, p = .118	r = .051, p = .118	r = .051, p = .118	r = .051, p = .118
Correlation min / max	r = -.272 / -.118	r = -.391 / .041	r = -.206 / .097	r = -.595 / .054	r = -.078 / -.003	r = -.078 / -.003	r = -.078 / -.003	r = -.078 / -.003	r = -.078 / -.003
Pairwise correlation differences / maximum possible	1 / 1	2 / 3	2 / 6	4 / 10	0 / 15	0 / 21	0 / 28	0 / 36	0 / 45
Significance of correlation differences	p = .021	p = 0	p = .001	p = .004	p = .986	p = .986	p = .986	p = .986	p = .986

[1]Possible cutoff for excessively small profiles (Spurk et al., 2020). [2]Indicators of possible variance-covariance misspecification.

Table S4

Sample Statistics for D Models (Unique Variances and Common Covariance) with 2–10 Profiles in Study 1 (*N* = 939)

	Number of Profiles								
	2	3	4	5	6	7	8	9	10
Estimation problems	No	No	No	No	Yes	Yes	Yes	Yes	Yes
Profiles containing less than 3% of sample[1]	0	0	1	1	1	2	3	4	5
Profiles containing less than 1% of sample[1]	0	0	0	0	0	2	3	3	4
Profiles containing fewer than 25 cases[1]	0	0	1	1	1	2	3	4	5
Empty profiles	0	0	0	0	0	2	2	3	4
Pairwise variance differences (material capital) / maximum possible[2]	1 / 1	3 / 3	5 / 6	9 / 10	13 / 15	9 / 21	9 / 28	14 / 36	14 / 45
Pairwise variance differences (social capital) / maximum possible[2]	1 / 1	1 / 3	4 / 6	4 / 10	11 / 15	9 / 21	9 / 28	10 / 36	10 / 45
Profiles with significant correlations	2	2	2	0	2	4	3	1	1
Mean correlation strength (weighted)	r = .192, p = 0	r = .154, p = 0	r = .12, p = 0	r = .022, p = .498	r = .147, p = 0	r = .244, p = 0	r = .206, p = 0	r = .097, p = .004	= .097, p = .00
Correlation min / max	r = -.297 / -.136	r = -.331 / -.093	r = -.176 / .107	r = -.121 / .097	r = -.205 / .097	r = -.278 / -.18	r = -.196 / 1	r = -.128 / .097	r = -.128 / .097
Pairwise correlation differences / maximum possible	1 / 1	1 / 3	2 / 6	0 / 10	0 / 15	0 / 21	0 / 28	0 / 36	0 / 45
Significance of correlation differences	0	0	1	1	1	2	3	4	5

[1]Possible cutoff for excessively small profiles (Spurk et al., 2020). [2]Indicators of possible variance-covariance misspecification. [3]Could not be computed with *mvtest* in Stata (Stata Corp., 2021) due to a singular correlation matrix. [4]Could not be computed with *mvtest* in Stata (Stata Corp., 2021) due to small classes.

Table S5

Sample Statistics for E Models (Unique Variances and Unique Covariances) with 2–10 Profiles in Study 1 (*N* = 939)

	Number of Profiles								
	2	3	4	5	6	7	8	9	10
Estimation problems	No	No	No	No	Yes	Yes	Yes	Yes	Yes
Profiles containing less than 3% of sample[1]	0	0	0	1	2	2	3	4	5
Profiles containing less than 1% of sample[1]	0	0	0	0	1	1	2	3	4
Profiles containing fewer than 25 cases[1]	0	0	0	1	2	2	3	4	5
Emptry profiles	0	0	0	0	1	1	2	3	4
Pairwise variance differences (material capital) / maximum possible[2]	1 / 1	3 / 3	5 / 6	8 / 10	8 / 15	14 / 21	14 / 28	14 / 36	14 / 45
Pairwise variance differences (social capital) / maximum possible[2]	1 / 1	3 / 3	5 / 6	4 / 10	7 / 15	10 / 21	10 / 28	10 / 36	10 / 45
Profiles with significant correlations	2	2	3	3	2	2	2	2	2
Mean correlation strength (weighted)	r = .234, p = 0	r = .215, p = 0	r = .398, p = 0	r = .335, p = 0	r = .345, p = 0	r = .137, p = 0	r = .137, p = 0	r = .137, p = 0	r = .137, p = 0
Correlation min / max	r = -.395 / -.152	r = -.435 / .107	r = -.763 / .132	r = -.643 / .122	r = -.697 / .103	r = -.205 / .097	r = -.205 / .097	r = -.205 / .097	r = -.205 / .097
Pairwise correlation differences / maximum possible	1 / 1	2 / 3	4 / 6	7 / 10	5 / 15	2 / 21	2 / 28	2 / 36	2 / 45
Significance of correlation differences	p = 0	p = 0	p = 0	p = 0	p = 0	p = .066	p = .066	p = .066	p = .066

[1]Possible cutoff for excessively small profiles (Spurk et al., 2020). [2]Indicators of possible variance-covariance misspecification.

Table S6

Sample Statistics for A Models (Equal Variances and No Covariance) with 2–10 Profiles in Study 2 (*N* = 2,408)

	Number of Profiles								
	2	3	4	5	6	7	8	9	10
Estimation problems	No	No	No	No	No	Yes	Yes	Yes	Yes
Profiles containing less than 3% of sample[1]	0	0	0	0	0	—[3]	0	1	1
Profiles containing less than 1% of sample[1]	0	0	0	0	0	—[3]	0	0	0
Profiles containing fewer than 25 cases[1]	0	0	0	0	0	—[3]	0	0	0
Empty profiles	0	0	0	0	0	—[3]	0	0	0
Pairwise variance differences (material capital) / maximum possible[2]	0 / 1	2 / 3	4 / 6	9 / 10	14 / 15	—[3]	16 / 28	23 / 36	32 / 45
Pairwise variance differences (social capital) / maximum possible[2]	1 / 1	2 / 3	4 / 6	4 / 10	6 / 15	—[3]	11 / 28	20 / 36	23 / 45
Profiles with significant correlations	0	1	0	1	1	—[3]	0	0	0
Mean correlation strength (weighted)	r = .025, p = .222	r = .067, p = 0	r = .05, p = .014	r = .058, p = .004	r = .038, p = .064	—[3]	r = .032, p = .114	r = .033, p = .106	r = .044, p = .03
Correlation min / max	r = .011 / .066	r = -.141 / -.005	r = -.033 / .079	r = -.061 / .124	r = -.062 / .124	—[3]	r = -.067 / .05	r = -.067 / .312	r = -.08 / .319
Pairwise correlation differences / maximum possible	0 / 1	1 / 3	0 / 6	2 / 10	2 / 15	—[3]	0 / 28	0 / 36	0 / 45
Significance of correlation differences	p = .276	p = .027	p = .201	p = .108	p = —[3]	—[3]	p = —[3]	p = —[3]	p = —[3]

[1]Possible cutoff for excessively small profiles (Spurk et al., 2020). [2]Indicators of possible variance-covariance misspecification. [3]Could not be computed with *mvtest* in Stata (Stata Corp., 2021) due to a singular correlation matrix.

Table S7

Sample Statistics for B Models (Equal Variances and Common Covariance) with 2–10 Profiles in Study 2 (N = 2,408)

	Number of Profiles								
	2	3	4	5	6	7	8	9	10
Estimation problems	No	No	No	No	No	Yes	Yes	Yes	Yes
Profiles containing less than 3% of sample[1]	0	0	0	0	0	—[3]	0	0	0
Profiles containing less than 1% of sample[1]	0	0	0	0	0	—[3]	0	0	0
Profiles containing fewer than 25 cases[1]	0	0	0	0	0	—[3]	0	0	0
Empty profiles	0	0	0	0	0	—[3]	0	0	0
Pairwise variance differences (material capital) / maximum possible[2]	0 / 1	2 / 3	4 / 6	9 / 10	15 / 15	—[3]	19 / 28	23 / 36	28 / 45
Pairwise variance differences (social capital) / maximum possible[2]	1 / 1	2 / 3	4 / 6	5 / 10	6 / 15	—[3]	11 / 28	12 / 36	13 / 45
Profiles with significant correlations	1	2	1	1	1	—[3]	0	0	0
Mean correlation strength (weighted)	r = .039, p = .052	r = .102, p = 0	r = .058, p = .004	r = .053, p = .008	r = .038, p = .064	—[3]	r = .032, p = .114	r = .033, p = .104	r = .035, p = .086
Correlation min / max	r = .02 / .096	r = -.18 / -.029	r = .011 / .189	r = -.053 / .124	r = -.062 / .124	—[3]	r = -.067 / .05	r = -.081 / .05	r = -.126 / .018
Pairwise correlation differences / maximum possible	0 / 1	1 / 3	2 / 6	2 / 10	2 / 15	—[3]	0 / 28	0 / 36	0 / 45
Significance of correlation differences	p = .137	p = .012	p = .064	p = .141	p = —[3]	—[3]	p = —[3]	p = —[3]	p = —[3]

[1]Possible cutoff for excessively small profiles (Spurk et al., 2020). [2]Indicators of possible variance-covariance misspecification. [3]Could not be computed with *mvtest* in Stata (Stata Corp., 2021) due to a singular correlation matrix.

Table S8

Sample Statistics for C Models (Unique Variances and No Covariance) with 2–10 Profiles in Study 2 (N = 2,408)

	Number of Profiles								
	2	3	4	5	6	7	8	9	10
Estimation problems	No	No	No	Yes	Yes	Yes	Yes	Yes	Yes
Profiles containing less than 3% of sample[1]	0	1	0	1	1	—[3]	3	5	—[3]
Profiles containing less than 1% of sample[1]	0	1	0	1	1	—[3]	3	5	—[3]
Profiles containing fewer than 25 cases[1]	0	1	0	1	1	—[3]	3	5	—[3]
Empty profiles	0	0	0	1	1	—[3]	3	4	—[3]
Pairwise variance differences (material capital) / maximum possible[2]	1 / 1	1 / 3	6 / 6	6 / 10	7 / 15	—[3]	7 / 28	5 / 36	—[3]
Pairwise variance differences (social capital) / maximum possible[2]	1 / 1	1 / 3	3 / 6	3 / 10	10 / 15	—[3]	10 / 28	6 / 36	—[3]
Profiles with significant correlations	2	2	3	3	1	—[3]	1	2	—[3]
Mean correlation strength (weighted)	r = .123, p = 0	r = .109, p = 0	r = .113, p = 0	r = .113, p = 0	r = .072, p = 0	—[3]	r = .072, p = 0	r = .094, p = 0	
Correlation min / max	r = -.138 / -.109	r = -1 / -.108	r = -.166 / .081	r = -.166 / .081	r = -.357 / .091	—[3]	r = -.357 / .091	r = -1 / .249	—[3]
Pairwise correlation differences / maximum possible	0 / 1	0 / 3	3 / 6	3 / 10	4 / 15	—[3]	4 / 28	3 / 36	—[3]
Significance of correlation differences	p = .523	p = —[3]	p = 0	p = 0	p = 0	—[3]	p = 0	p = —[3]	—[3]

[1]Possible cutoff for excessively small profiles (Spurk et al., 2020). [2]Indicators of possible variance-covariance misspecification. [3]Estimation failed.

Table S9

Sample Statistics for D Models (Unique Variances and Common Covariance) with 2–10 Profiles in Study 2 (*N* = 2,408)

	Number of Profiles								
	2	3	4	5	6	7	8	9	10
Estimation problems	No	No	No	Yes	Yes	Yes	Yes	Yes	Yes
Profiles containing less than 3% of sample[1]	0	0	0	1	2	—[3]	3	—[3]	—[3]
Profiles containing less than 1% of sample[1]	0	0	0	1	2	—[3]	3	—[3]	—[3]
Profiles containing fewer than 25 cases[1]	0	0	0	1	2	—[3]	3	—[3]	—[3]
Empty profiles	0	0	0	1	2	—[3]	3	—[3]	—[3]
Pairwise variance differences (material capital) / maximum possible[2]	1 / 1	3 / 3	6 / 6	6 / 10	6 / 15	—[3]	7 / 28	—[3]	—[3]
Pairwise variance differences (social capital) / maximum possible[2]	1 / 1	2 / 3	3 / 6	3 / 10	3 / 15	—[3]	10 / 28	—[3]	—[3]
Profiles with significant correlations	2	1	2	2	2	—[3]	1	—[3]	—[3]
Mean correlation strength (weighted)	r = .181, p = 0	r = .085, p = 0	r = .103, p = 0	r = .103, p = 0	r = .103, p = 0		r = .061, p = .002		
Correlation min / max	r = -.195 / -.166	r = -.03 / .13	r = -.167 / .081	r = -.167 / .081	r = -.167 / .081	—[3]	r = -.378 / .035	—[3]	—[3]
Pairwise correlation differences / maximum possible	0 / 1	1 / 3	3 / 6	3 / 10	3 / 15	—[3]	4 / 28	—[3]	—[3]
Significance of correlation differences	p = .508	p = .026	p = 0	p = 0	p = 0	—[3]	p = 0	—[3]	—[3]

[1]Possible cutoff for excessively small profiles (Spurk et al., 2020). [2]Indicators of possible variance-covariance misspecification. [3]Estimation failed.

Table S10

Sample Statistics for E Models (Unique Variances and Unique Covariances) with 2–10 Profiles in Study 2 (*N* = 2,408)

	Number of Profiles								
	2	3	4	5	6	7	8	9	10
Estimation problems	No	No	Yes	Yes	Yes	Yes	Yes	Yes	Yes
Profiles containing less than 3% of sample[1]	0	0	0	1	2	—[3]	—[3]	—[3]	—[3]
Profiles containing less than 1% of sample[1]	0	0	0	0	2	—[3]	—[3]	—[3]	—[3]
Profiles containing fewer than 25 cases[1]	0	0	0	0	2	—[3]	—[3]	—[3]	—[3]
Empty profiles	0	0	0	0	2	—[3]	—[3]	—[3]	—[3]
Pairwise variance differences (material capital) / maximum possible[2]	1 / 1	3 / 3	6 / 6	10 / 10	6 / 15	—[3]	—[3]	—[3]	—[3]
Pairwise variance differences (social capital) / maximum possible[2]	1 / 1	2 / 3	3 / 6	8 / 10	5 / 15	—[3]	—[3]	—[3]	—[3]
Profiles with significant correlations	2	1	2	4	1	—[3]	—[3]	—[3]	—[3]
Mean correlation strength (weighted)	r = .189, p = 0	r = .11, p = 0	r = .149, p = 0	r = .153, p = 0	r = .067, p = .002				
Correlation min / max	r = -.222 / -.156	r = -.062 / .164	r = -.224 / .167	r = .031 / .587	r = .003 / .131	—[3]	—[3]	—[3]	—[3]
Pairwise correlation differences / maximum possible	0 / 1	3 / 3	3 / 6	7 / 10	0 / 15	—[3]	—[3]	—[3]	—[3]
Significance of correlation differences	p = .131	p = .001	p = 0	p = 0	p = .369	—[3]	—[3]	—[3]	—[3]

[1]Possible cutoff for excessively small profiles (Spurk et al., 2020). [2]Indicators of possible variance-covariance misspecification. [3]Estimation failed.

Table S11

Correlations Between Profile Indicators in Study 1

	1	2	3
1. Income	1	-0.02	0.39*
2. Social Support		1	-0.02
3. Education			1

*Denotes significance at p<.05

Table S12

Correlations Between Profile Indicators in Study 2

	1	2	3
1. Income & Assets	1	0.37*	0.13*
2. Education		1	0.11*
3. Social Support			1

* Denotes significance at p<.05

Table S13

Model Fit (BIC) for Latent Profile Models in Study 1; Preferred Model (3E) in bold

Variance–covariance structure	Number of profiles									
	1	2	3	4	5	6	7	8	9	10
A. Equal variances; no covariance	367	144	113	83	71	57	54	69	90	–
B. Equal variances; single covariance	373	149	117	88	72	63	60	75	–	–
C. Unique variances; no covariance		112	84	73	–	–	–	–	–	–
D. Unique variances; single covariance		112	76	80	78	–	–	–	–	–
E. Unique variances; unique covariances		111	**76**	73	–	–	–	–	–	–

Note. Dashes indicate models omitted due to estimation problems.

Table S14

Classification Table for Preferred Solution (3E) in Study 1

		Mean probability of profile membership		
Class Group	*n*	1	2	3
1. The Underclass	310	**.88**	.02	.10
2. Vulnerable Workers	173	.12	**.69**	.19
3. Middle/Upper Class	456	.10	.06	**.84**

Note. Most likely profile membership in bold.

Table S15

Model Fit (BIC) for Latent Profile Models in Study 2; Preferred Model (4C) in bold

Variance–covariance structure	Number of profiles									
	1	2	3	4	5	6	7	8	9	10
A. Equal variances; no covariance	2890	2262	2253	1864	1698	1103	–	–	–	–
B. Equal variances; single covariance	2863	2268	2260	1869	1705	1111	–	–	–	–
C. Unique variances; no covariance		2203	2239	**1601**	–	–	–	–	–	–
D. Unique variances; single covariance		2206	1697	1608	–	–	–	–	–	–
E. Unique variances; unique covariances		2213	1696	–	–	–	–	–	–	–

Note. Dashes indicate models omitted due to estimation problems.

Table S16

Classification Table for Preferred Solution (4C) in Study 2

		Mean probability of profile membership			
Class Group	*n*	1	2	3	4
1. The Underclass	797	**.81**	.11	.04	.04
2. Vulnerable Workers	316	.24	**.64**	.05	.07
3. Secure Workers	445	.05	.14	**.80**	.01
4. Middle/Upper Class	850	.02	.09	.02	**.87**

Figure S1

Log-likelihood and BIC graphs for all models in Study 1 (N = 939)

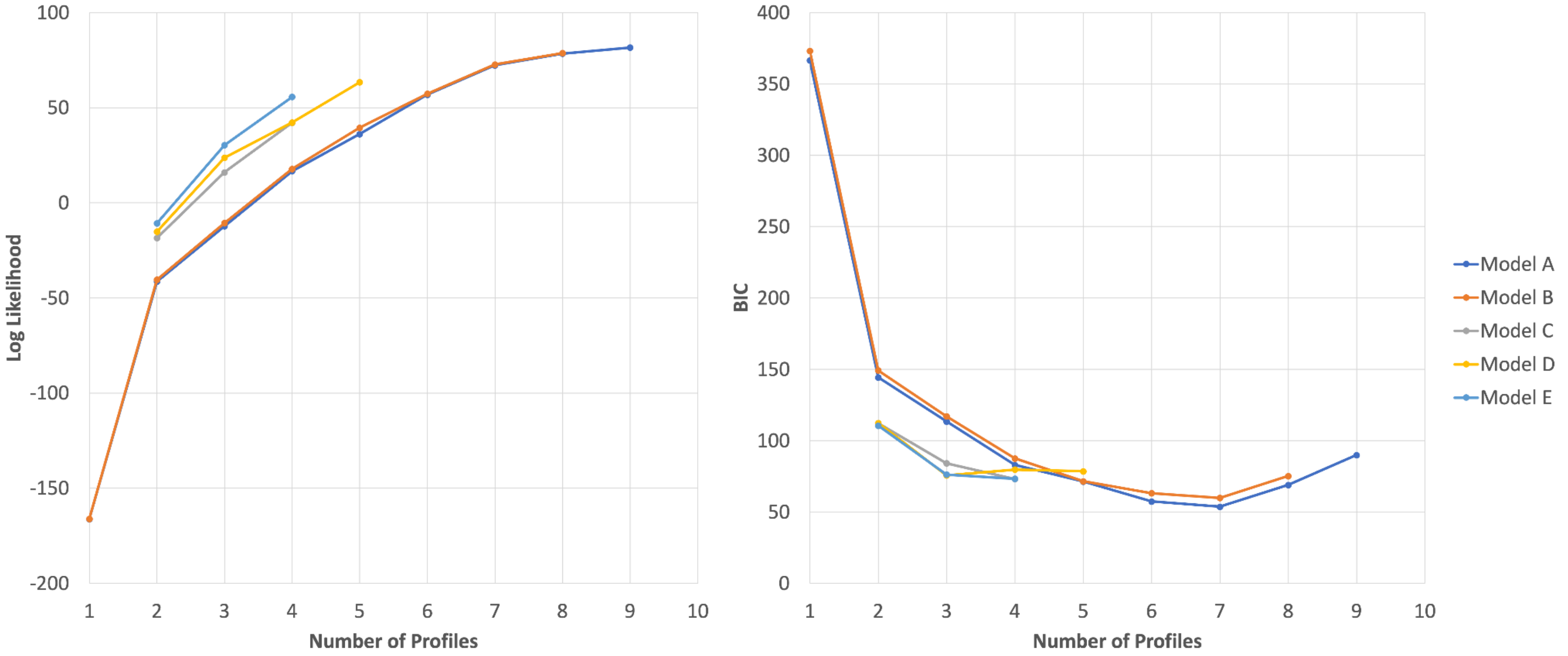

Figure S2

Log-likelihood and BIC graphs for all models in Study 2 (N = 2,408)

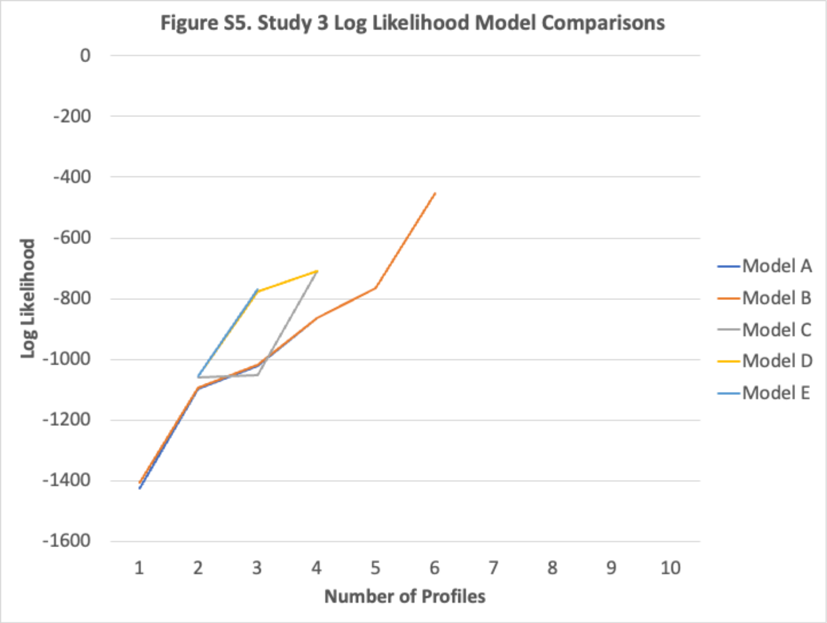

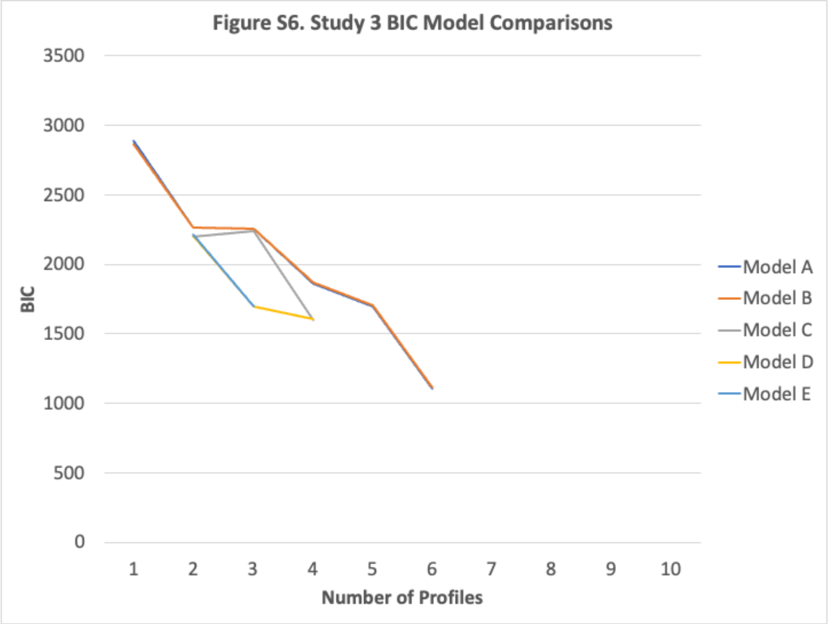

Figure S3

Plots depicting the nonlinear associations identified by the generalized additive models (GAMs) between income/household assets and social support in Study 1 (left panel) and Study 2 (right panel).

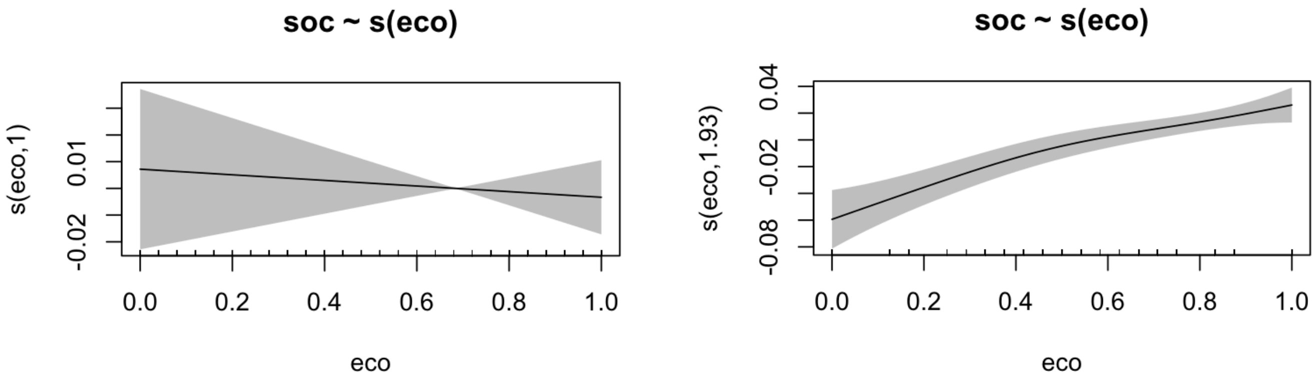

Supplement: Supplementary file 1 [file Data_Sheet_1.PDF]
